# Obesity: Friend or Foe in Sjögren’s Syndrome Patients?

**DOI:** 10.3390/diagnostics14232725

**Published:** 2024-12-03

**Authors:** Kincső Mezei, Laura Nagy, Viktória Orosz, Zsófia Aradi, Bernadett Bói, Antónia Szántó

**Affiliations:** 1 Division of Clinical Immunology, Institute of Internal Medicine, Faculty of Medicine, University of Debrecen, H-4032 Debrecen, Hungary; mezei.kincso@med.unideb.hu (K.M.);; 2 Gyula Petrányi Clinical Immunology and Allergology Doctoral School, University of Debrecen, H-4032 Debrecen, Hungary; 3 Department of Public Health and Epidemiology, Faculty of Medicine, University of Debrecen, H-4032 Debrecen, Hungary

**Keywords:** Sjögren’s syndrome, obesity, statin, obesity paradox

## Abstract

Background/Objectives: In Sjögren’s syndrome, exocrine glands are destructed in an autoimmune-mediated process. Obesity is known to influence a wide range of diseases. This study aimed to examine whether obesity has an impact on the disease course of our patients with Sjögren’s syndrome. Methods: Out of the regularly followed-up patients, 125 were grouped based on their body mass index (BMI). Below a BMI of 25, they were listed as “non-obese” (*n* = 45), whereas above a BMI of 25, they were categorized as “obese” (*n* = 80). Demographic, laboratory, and immunological parameters; Sjögren’s syndrome disease activity index; certain extraglandular manifestations; and treatment modalities were compared using biostatistical methods. Results: Among the examined cardiovascular and cerebrovascular co-morbidities, type 2 diabetes and hypertension were significantly more frequent in the obese group. Considering the associated further autoimmune disorders and extraglandular manifestations, in our patients, there were no significant differences between the two groups. Among laboratory parameters, gamma glutamil transferase, alanine transaminase, hemoglobin, hematocrit, lymphocyte rate, triglyceride, and c3 and c4 complement levels were significantly higher in the obese group, while the proportion of rheumatoid factor positivity and the neutrophil granulocyte rate were significantly lower. Immunoglobulin G, A, and M levels did not differ significantly between the two subsets. Obese patients needed steroid therapy significantly less frequently; however, statin therapy was remarkably more frequent in that group. Furthermore, the European League Against Rheumatism (EULAR) Sjögren’s syndrome disease activity index (ESSDAI) was significantly lower in the group of overweight patients. Conclusions: Our results suggest that several immunological parameters of obese patients are more favorable compared to those with normal body weight. Behind that, we might suspect either the beneficial effect of statin therapy and/or the obesity paradox.

## 1. Introduction

In Sjögren’s syndrome (SS), ductal epithelial cells are destructed in an autoimmune-mediated process, but the disease may also cause various extraglandular manifestations, such as non-erosive polyarthritis, central and peripheral nervous system manifestations, interstitial lung disease, muscular involvement, nephropathy, vasculitis, and different types of autoimmune cytopenia, among others. Diagnosis of SS should be based on the usual screening questions (has the patient been experienced dry eyes and/or dry mouth for more than 3 months) and basic evaluation tests. Moreover, depending on the complaints and manifestations, further examination methods could be necessary (ultrasound, X-ray, computer tomography (CT), electroneuro/myography etc.). Basic evaluation tests include unstimulated salivary flow rate, Schirmer’s test, lissamine green or fluorescein green test, antinuclear antibody (ANA) immunofluorescence, ANA titer, anti-Ro/SS-A titer. More than 80% of patients with SS test positive for ANA, 40–75% for anti-Ro/SS-A, and 23–52% for anti-La/SS-B. Positive autoantibodies could indicate early-onset disease and more intense tissue infiltration, and are associated with a higher prevalence of extraglandular manifestations. Labial salivary gland biopsy is a minimal invasive examination which screens for focal periductal infiltration of dominant clusters of differentiation (CD)4+ lymphocytes, with fewer CD8+ T-cells and CD19+ B-cells, some plasma cells, and dendritic cells [1,2].

Obesity is a rather serious worldwide issue known to significantly influence numerous diseases. In obese patients, adipose tissue secretes various compounds, which induce and sustain inflammation. This proinflammatory state of visceral adipose tissue is believed to accelerate cardiovascular and metabolic diseases in overweight patients [3]. According to the World Health Organization (WHO), obesity is defined as abnormal fat accumulation. Obesity is diagnosed by body mass index (BMI), regardless of age and gender, and it is categorized in different classes in accordance with BMI: normally, this parameter is under 25, whereas overweight is defined as BMI between 25 and 30, and if BMI is above 30, it is categorized as obesity. It is important to note, that there are several types of obesity: not all overweight patients are necessarily considered unhealthy. Metabolically healthy individuals can present with perfect parameters, regardless of their weight. This obesity paradox is frequently studied in cardiovascular diseases, since in a high number of cases, survival and prognosis of obese patients have proved to be better [3]. In both obesity and autoimmune diseases, it is important to consider lipid levels, especially because of accelerated atherosclerosis, which is partly caused by the side effects of treatment (e.g., corticosteroids), but also triggered by autoinflammatory mechanisms. Immune cells can be found in atherosclerotic plaques, which suggests that they have specific roles in their generation by migrating into and activating inside them. This could be triggered by various stimuli. Experiments suggest that CD4+ and CD8+ T-cells have a role in forming atherosclerotic plaques and accelerating atherosclerosis. These also participate in creating inflammation and secreting various cytokines, including interleukins and tumor necrosis factor alpha. Autoantibodies help with the formation of atherosclerotic plaques as well; however, their significance can be different in each autoimmune disease. SS patients are believed to have lower levels of anti-lipoprotein lipase compared to other systemic autoimmune diseases, but of course, this hypothetical benefit could be lost by having hyperlipidemia and/or obesity [4]. Hyperlipidemia can be classified as isolated elevation of cholesterol levels, elevated triglyceride levels, or elevation of both parameters among laboratory values. As for etiology, it could be caused by hereditary factors, but is more likely an acquired, environmental problem in most patients. Hyperlipidemia is one of the main risk factors for cardiovascular diseases, which are amongst the leading causes of death. It is easily diagnosed by a lipid screen panel, and with correct therapy, lipid levels can be altered very successfully [5].

Originally, the idea of this research came from real-life practice. Over the years, we have noticed that thin people suffering from SS usually have more complaints and it is more challenging to manage their disease, while overweight SS patients seem to cope better with their illness. Therefore, our study was carried out to determine how our patients’ overweight affects the disease course of their SS compared to non-obese SS patients.

## 2. Materials and Methods

An a priori power analysis was conducted using G*Power version 3.1.9.7 (Heinrich Heine Universität, Düsseldorf, Germany) to determine the minimum sample size for the two groups (80% power, detecting medium effect, α = 0.05).

We examined the data of 125 consecutive patients who were regularly followed up with in our outpatient clinic (University of Debrecen, Faculty of Medicine, Division of Clinical Immunology, Debrecen, Hungary). SS was diagnosed according to the American College of Rheumatology/European League Against Rheumatism (ACR/EULAR criteria). This is based on the summed score of five items: anti-Ro/SS-A antibody positivity and focal lymphocytic sialadenitis with a focus score ≥ 1 foci/mm^3^, which both score 3 points; abnormal ocular staining score, with five or more points (or van Bijsterveld score with more than 4 point); positive Schirmer’s test (≤5 mm/5 min); and a decreased unstimulated salivary flow rate equal or less than 0.1 mL/min, which are worth 1 point each. Patients who have complaints suggestive of SS with a total score ≥ 4 for the items above can be classified as having SS. The sensitivity and specificity of the ACR/EULAR 2016 criteria are quite high, with 96% [6].

BMI was calculated for each patient to categorize them into two subgroups. Patients with BMI < 25 were categorized as “non-obese” (*n* = 45, out of which 43 were women and 2 were men), and patients with BMI ≥ 25 were listed into the “obese” group (*n* = 80, out of which 75 were women and 5 were men)—however, by definition, this category merged overweight and obese patients. Cardiovascular risk factors (co-existing hypertension, type 2 diabetes mellitus, stroke, ischemic heart disease, and carotid artery stenosis), presence of other systemic or organ specific autoimmune diseases (e.g., rheumatoid arthritis (RA), systemic lupus erythematosus (SLE), antiphospholipid syndrome, Raynaud’s phenomenon, or Hashimoto thyroiditis), extraglandular symptoms (such as polyarthritis, pulmonary manifestations, nephropathy, cryoglobulinemia, and neuromuscular or cutaneous manifestations), and ongoing therapy regarding glucocorticoids, immunosuppressives and statins were also compared.

Among laboratory parameters, liver and kidney function tests, aspartate transaminase (AST), alanine transaminase (ALT), gamma glutamil transferase (GGT), alkaline phosphatase (AP), lactate dehydrogenase (LDH), glomerular filtration rate (GFR), urea, creatinine levels, hematological parameters (leukocyte count, proportion of lymphocytes, neutrophil granulocytes, hemoglobin levels, platelet counts), erythrocyte sedimentation rate (ESR), and immune serological markers (presence of anti-Ro/SS-A, anti-La/SS-B, rheumatoid factor, serum immunoglobulin (Ig)A, IgG, IgM and complement levels (c3, c4, 50% hemolytic complement activity (CH50)) were compared. EULAR Sjögren’s Syndrome Disease Activity Index (ESSDAI) was also calculated for patients who did not have a symptom shared with an associated systemic autoimmune disorder, which might have interfered with the result.

Values were organized into tables, and then specific biostatistical methods were applied to determine their statistical significance. Statistical Package for Social Sciences (SPSS) (PASW Statistics for Windows, version 18.0. SPSS Inc., Chicago, IL, USA) software was used for statistical analysis, and GraphPad Prism (GraphPad Prism for Windows, version 10.3.1, Boston, MA, USA) software was used for the graphical presentation. The Kolmogorov–Smirnov test was used for the evaluation of normality. For continuous parameters not showing a normal distribution, the Mann–Whitney test was carried out, whereas for those with normal distribution, the two-sample *t*-test was used. For discrete parameters, Fisher’s exact test was used when the expected count was <5.00, while the chi-square test was performed when the expected data were >5.00. “*p*” values < 0.05 were considered statistically significant.

Multiple linear regression was carried out to investigate the relationship between obesity (predictor) and ESSDAI (dependent).

All parameters were in accordance with the standards of the Local Ethical Committee and the Declaration of Helsinki.

Informed consent was obtained from each patient.

## 3. Results

### 3.1. Cardiovascular Diseases

Among the examined metabolic, cardiovascular, and cerebrovascular co-morbidities, the occurrence of type 2 diabetes and hypertension was significantly more frequent in the obese group, as was expected. However, no significant difference was found regarding the occurrence of stroke, carotid artery stenosis, or ischemic heart disease (Table 1).

### 3.2. Autoimmune Disorders and Extraglandular Manifestations

Considering the co-occurring autoimmune disorders besides SS, in our patients, there was no significant difference between the two groups regarding the prevalence of systemic autoimmune disorders such as RA, SLE, and antiphospholipid syndrome. Since there were several patients with one or more co-existing systemic autoimmune disorders, the cumulative number of patients with associated SS was 18 (22.5%) in the obese group and 12 (26.6%) in the non-obese group, which did not show significant differences either. The occurrence of Hashimoto thyroiditis and Raynaud’s phenomenon did not differ significantly between the two groups, either (Table 2).

**Figure 1:**
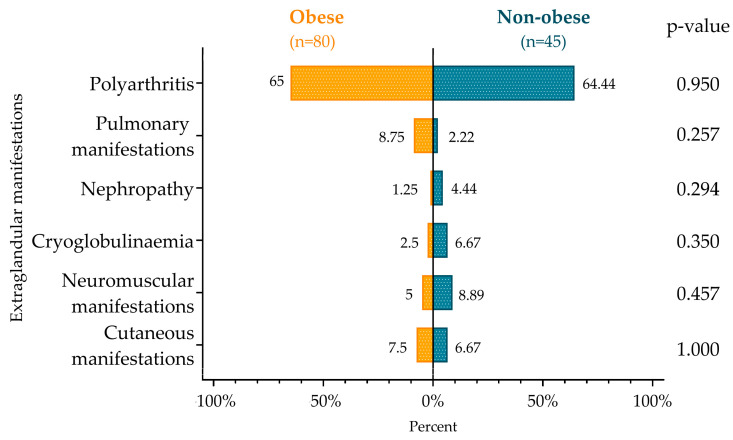
Prevalence of extraglandular manifestations in the obese and non-obese groups.

Similarly, no significant difference was detected in the occurrence of extraglandular manifestations, such as skin conditions (e.g., purpura, erythema multiforme, subacute cutaneous lupus erythematosus, different types of vasculitis), neuromuscular manifestations (myositis, peripheral or central nervous system involvement), cryoglobulinemia, renal involvement, pulmonary manifestations, or polyarthritis in the two patient groups (Figure 1).

### 3.3. Laboratory Parameters and Immune Serological Results

Among laboratory parameters, a remarkable difference was found between GGT, AP, and triglyceride levels, which were—as expected—all higher in the obese group. No significant difference was found between GFR, urea, creatinine, AST, ALT, CRP, LDH, cholesterol levels, leukocyte count, platelet count, and ESR. Hemoglobin levels were significantly lower in the subset of patients with normal body weights. In addition, the proportion of lymphocytes was significantly higher, whereas the proportion of neutrophil granulocytes was significantly lower in overweight SS patients compared to non-obese ones (Table 3). However, it should be noted that all the above-mentioned values were within the normal range.

Considering immune serological results, obese patients had slightly higher IgM and lower IgG and IgA levels, although all their medians were within the normal range (Table 3). Less obese patients had anti-Ro/SS-A and anti-La/SS-B positivity, although these differences were not significant. The proportion of patients with rheumatoid factor positivity was significantly lower in the obese group. Regarding complement levels, both c3 and c4 proved to be significantly higher in the obese group (Table 3).

### 3.4. Disease Management and Disease Activity

According to our observations, obese patients required steroid therapy significantly less often than the non-obese ones. A disease-modifying antirheumatic drug (DMARD) requirement was more common, although not significantly, in the non-obese group. On the other hand, statin use was significantly more frequent in the group of obese patients (Table 4). ESSDAI was calculated in most patients (65/80 in obese and 39/45 in non-obese ones). As was previously mentioned, patients with associated autoimmune disorders who had symptoms which might have interfered with their co-existing autoimmune disease were not included in the ESSDAI calculation. Moreover, outliers (3rd quartile + 1.5x interquartile range and 1st quartile − 1.5x interquartile range) were removed for easier interpretation. This affected four obese patients (ESSDAI 24, 19, 13, and 12, respectively) and one non-obese patient (ESSDAI 17) suffering from severe pulmonary (*n* = 2—ESSDAI 24 and 19), or moderate nephrological (*n* = 1, ESSDAI 13), muscular (*n* = 1, ESSDAI 12), or peripheral nervous system (*n* = 1, ESSDAI 17) manifestations.

**Figure 2:**
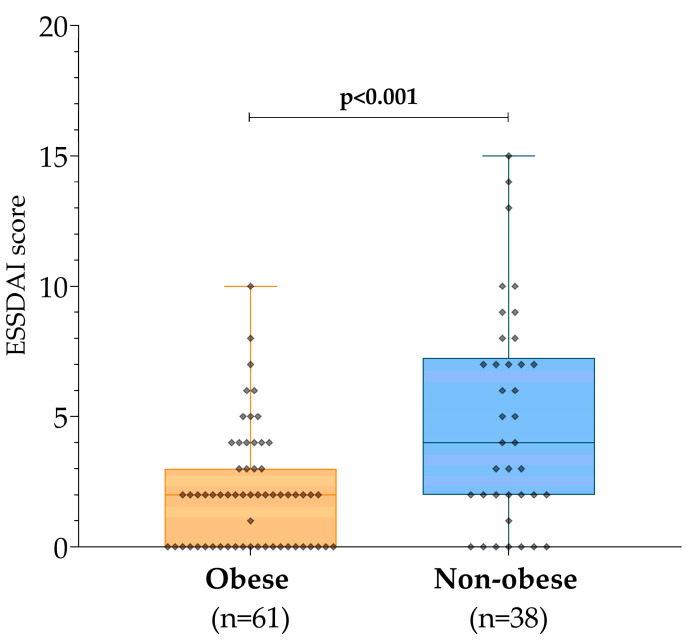
EULAR Sjögren’s syndrome disease activity index (ESSDAI) in the obese and non-obese groups. Abbreviation: EULAR: European League Against Rheumatism.

Thus, in our cohort, the median ESSDAI in the obese group was 2 (0–3, *n* = 61), significantly lower than in the non-obese patients, where the median score was 4 (2–7, *n* = 38) (*p* < 0.001; Figure 2).

### 3.5. Multiple Linear Regression Analysis

A multiple linear regression was run to predict ESSDAI according to obesity, steroid treatment, statin treatment, anti-Ro/SS-A+, anti-La/SS-B+, rheumatoid factor+, CRP (mg/L), LDH (U/L), triglyceride (mmol/L) and cholesterol (mmol/L). This resulted in a significant model: F (10, 91) = 3.507, *p* < 0.001, R^2^ = 0.278. ESSDAI was significantly associated with steroids (β = 0.346, *p* = 0.001) and LDH (β = 0.235, *p* = 0.013), but not with obesity, although there was a trend towards inverse association (β = −0.172, *p* = 0.099) (Table 5).

## 4. Discussion

Obesity is far more than only a cardiovascular risk factor. It might contribute to the development of several further pathological conditions [3,5]. This is why the results of our paper are surprising, since in our patients, obesity seems to have a protecting feature, leading to a more beneficial disease course with better parameters and lower disease activity index. The explanation behind this finding is quite complex, with several potential contributing factors, such as the protective effect of statins and/or the obesity paradox.

Another interesting finding was that in the overweight patients, the neutrophil rate was lower and the lymphocyte rate was higher, even though it is conventionally the other way around in autoimmune disorders [7,8].

In SS, there are two main elements of disease management: glandular symptoms require substitution therapy to replace the missing glandular products, and if extraglandular symptoms arise, systemic immunomodulant treatment is required. Glucocorticoids are needed if cutaneous, pulmonary, musculoskeletal, or neurological manifestations develop. If glucocorticoid therapy is not sufficient or the dose tapering is not tolerated by the patient, DMARDs can be introduced. Amongst them, chloroquine or hydroxychloroquine treatment can be beneficial in case of arthralgias, myalgias, or certain skin manifestations, like subacute cutaneous lupus. If musculoskeletal pain or arthritis is present, methotrexate should be initiated, but leflunomide, sulfasalazine, or cyclosporin-A can be considered as well. Azathioprine could be administered for numerous extraglandular involvements, for instance, interstitial pneumonitis, autoimmune hepatitis, certain skin conditions, etc. Another option is mycophenolate mofetil, which can help in the case of interstitial lung disease or kidney manifestations [2,9,10]. Our findings that corticosteroid use was significantly less frequent among obese patients, as well as the fact that there was a tendency towards less DMARD use, indicate a more benign disease course in the obese population. Moreover, according to the results of the multiple linear regression model, corticosteroid use seems to be a factor which might significantly contribute to higher ESSDAI, although the relationship can also be the inverse, as patients with higher activity are more likely to receive glucocorticoid treatment.

Statins are known to have both anti-inflammatory and immunomodulatory features. They can inhibit the infiltration of immune cells into the atherosclerotic plaques by reducing the production of inflammatory markers and lowering the levels of circulating pro-inflammatory cytokines, for example, serum amyloid A or CRP and various interleukins, by blocking certain intracellular pathways; consequently, they reduce T-cell activation and macrophage infiltration into the vessel walls. This way, they are able to reduce the risk of development and/or recurrence of cardiovascular events [11,12,13,14]. Statins are commonly used in the management of overweight people, even if they do not suffer from any kind of autoimmune disorder, in order to lower the risk of cardiovascular diseases by altering lipid levels and therefore eliminating dyslipidemia. Statins are believed to have an anti-inflammatory effect, as well [15,16]. This group of medication was significantly more frequently administered in the obese group in the case of our patients, as shown previously. These above-mentioned effects of statins clearly seem to have a positive influence on the disease course of patients suffering from SS. Our results indicate that in the group of obese patients, where statin use was significantly more frequent, the ESSDAI score was significantly lower, suggesting a more beneficial disease course for overweight and obese patients. Less steroid and disease-modifying antirheumatic drug demand seems to be a secondary consequence of the above-mentioned factors. On the other hand, according to the results of our linear regression analysis, statins did not seem to directly influence ESSDAI.

In addition to our results highlighting that statins might have a positive effect during the management of SS, their role was investigated in several other systemic autoimmune diseases. Among them, the most convincing result was shown in RA, where the anti-inflammatory features of statins resulted in a significantly lower disease activity score (DAS28). Furthermore, several studies have concluded that the mortality of RA patients using statin therapy was remarkably lower than that of the patients who had RA but were not administered any kind of statin. According to their conclusions, statin therapy has a beneficial effect not only on disease activity and mortality, but also on the swollen joint count and the endothelial function and stiffness, therefore supporting the hypothesis of routine statin use in patients diagnosed with RA. Obviously, the potential side effects and contraindications of statin treatment must not be overlooked, and these drugs always should be introduced with caution, with the long-term risk–benefit ratio being taken into consideration as well [13,17,18,19,20]. Moreover, in another paper, discontinuation of statin therapy was associated with a higher risk of mortality in RA patients [21]. Regarding SLE, a meta-analysis concluded that, although statins did not have a direct effect on lupus activity, they did result in CRP reduction, thus achieving indirect beneficial effects on patients while also decreasing the effect of the well-known accelerated atherosclerosis in patients with SLE [22]. In a population-based study, initiation of statin therapy reduced overall mortality in several systemic autoimmune and rheumatological diseases, including SS, as well. However, this study did not focus on the effect of statins on the disease activity [23].

Another interesting explanation for our results could be the so called “obesity paradox”, although it has not really been studied in autoimmune disorders, which makes this possibility even more intriguing. According to this observation, overweight patients with a healthy metabolic state are supposed to have lower levels of circulating inflammatory cytokines, resulting in more favorable parameters and overall condition compared to the metabolically “unhealthy” obese ones. Moreover, adults with obesity but without comorbidities are considered to be healthier than lean persons with cardiovascular risk factors or certain types of comorbidities. Recent studies suggest that previous cardiovascular medications, obesity duration, and smoking status play an important role in the obesity paradox, as well. The “protective” effect of overweight or obesity has mainly been reported in cardiovascular diseases, but according to other studies, previous evidence has been found in cases of certain malignant diseases, chronic kidney disease, obstructive pulmonary disease, stroke, pulmonary hypertension, osteoporosis, critical illness, and sepsis. Undoubtedly, being obese and metabolically healthy has its benefits; however, this condition is limited to certain phenotypes of obesity. As for phenotypes, non-sarcopenic obesity can be characterized by increased fat tissue and increased lean mass, whereas athlete’s phenotype mainly consists of increased lean mass, which still elevates the BMI, and sarcopenic obesity means less lean mass and significantly more fat tissue accumulation. Usually, the first two phenotypes are the healthier forms, despite their weight [3,24]. Cardiological data have also confirmed that in the obesity paradox, cardioprotective medications—such as statin—play an important role. Obese patients with preceding statin therapy have lower mortality and better outcome measures than statin non-users [25]. Moreover, regarding autoimmune patients, a recent systematic review has confirmed that, in the case of patients with systemic autoimmune diseases, the probability of cardiovascular events (especially acute myocardial infarction, coronary revascularization, or stroke) and cardiovascular mortality is significantly higher compared to the general population [26]. Low-grade inflammation in autoimmune disorders or obesity can provoke vascular responses through the action of neutrophil extracellular traps, which play an important role in the inflammatory response associated with atherosclerosis and atherosclerotic plaque rupture, resulting in acute cardiovascular events. Patients may benefit from the introduction of certain imaging techniques, like carotid Doppler ultrasound, in primary prevention to diagnose early, mild atherosclerosis and initiate statin therapy in time to prevent further plaque formation, while also taking advantage of the anti-inflammatory effect of these drugs [26,27,28,29,30]. This is where our results are connected, and that is why we suppose that not only obesity alone, but also the related statin therapy, might contribute to a more favorable effect in the management of SS.

Of course, we have to admit that our paper has some limitations. Since the idea of this comparison came from everyday practice, detailed lipid fractions were not examined. Moreover, there are a few patients whose latest laboratory results were not completely available at the time of recruitment. Another limitation is the retrospective nature of the examination. However, further investigations are planned to clarify our initial observations more precisely. Therefore, the examination of certain adipokines secreted by fat tissue is planned to investigate whether they have any specific effect that could be related to a more beneficial disease course for obese patients.

## 5. Conclusions

Based on our results, several laboratory and immunological parameters, and ESSDAI altogether, the disease course is more beneficial in the case of obese SS patients compared to their fellow patients with normal body weight. Naturally, our results should not encourage anyone to gain weight in order to ease their experience of living with SS. Revising our findings, we believe that at least one reason behind these results could be statin therapy, which is known to have several immunomodulant and anti-inflammatory features. Systemic autoimmune diseases in general, and SS in particular, have been proven to increase the risk of premature mortality, which is fairly supported by the risk of cardiovascular consequences of chronic inflammation, like dyslipidemia. What we would like to emphasize is that, despite being obese, the unfavorable consequences of obesity can be at least partially reversed. Nevertheless, it is important to consider the metabolic state and comorbidities not only of overweight, but of lean patients, too, since they play a significant role in the outcome of their disease. Therefore, screening for lipid parameters and even using imaging techniques early on may help in introducing lipid-lowering treatments at the right time, which is of high importance and may have additional beneficial effects in the long-term management of patients with SS.

## Figures and Tables

**Table 1:** Prevalence of cardiovascular diseases in the obese and non-obese groups.

	Obese *n* (% of Total 80)	Non-Obese, *n* (% of Total 45)	*p*-Value	Effect-Size (w)
Hypertension	55 (68.75)	21 (46.67)	0.015	0.217
Type 2 Diabetes	14 (17.5)	1 (2.22)	0.012	0.226
Stroke	4 (5)	1 (2.22)	0.653	0.040
Ischemic heart disease	23 (28.75)	9 (20)	0.282	0.096
Carotid artery stenosis	5 (6.25)	2 (4.44)	1.000	0.000

**Table 2:** Prevalence of autoimmune disorders in the obese and non-obese groups.

	Obese *n* (% of Total 80)	Non-Obese, *n* (% of Total 45)	*p*-Value	Effect-Size (w)
Rheumatoid Arthritis	15 (18.75)	7 (15.56)	0.653	0.040
Systemic Lupus Erythematosus	4 (5)	4 (8.89)	0.457	0.067
Antiphospholipid Syndrome	3 (3.75)	3 (6.67)	0.666	0.039
Associated Sjögren’s syndrome	18 (22.5)	12 (26.6)	0.601	0.047
Hashimoto Thyreoiditis	11 (13.75)	6 (13.33)	0.948	0.006
Raynaud’s phenomenon	20 (25)	18 (40)	0.080	0.157

**Table 3:** Laboratory parameters in the obese and non-obese groups.

	Obese		Non-Obese		*p*-Value	Effect-Size
	Valid *n*	Median (IQR) or Mean ± SD or *n* (%)	Valid *n*	Median (IQR) or Mean ± SD or *n* (%)		
GFR (mL/p/1.73 m^2^)	79	80 (68–90)	45	79 (67–90)	0.989	d = 0.002
Urea (mmol/l)	79	5.1 (4.05–6.4)	45	4.9 (4.4–5.8)	0.419	d = 0.146
Creatinine (μmol/l)	79	69 (60.5–78)	45	71 (62–81)	0.582	d = 0.099
AST (U/L)	80	21 (17–26)	44	20.5 (16–25)	0.364	d = 0.164
ALT (U/L)	80	71.5 (58.5–91.25)	45	64 (56–73)	0.093	d = 0.305
GGT (U/L)	80	24 (17.75–47.5)	45	19 (15–25)	0.005	d = 0.523
AP (U/L)	80	21 (15–29.5)	45	16 (13–22)	0.010	d = 0.477
CRP (mg/l)	80	2.8 (1.5–6.6)	44	2.28 (1–7.1)	0.522	d = 0.115
LDH (U/L)	79	211 (188–244.5)	44	205.5 (178.75–233.75)	0.348	d = 0.169
Cholesterol (mmol/L)	79	5.02 ± 1.21	45	4.75 ± 1.15	0.231	d = 0.225
Triglyceride (mmol/L)	79	1.4 (1.05–1.95)	45	1.1 (0.7–1.4)	<0.001	d = 0.699
Leukocyte (G/L)	79	6.47 (5.3–7.84)	45	6.51 (5.42–8.72)	0.647	d = 0.082
Hemoglobin (g/l)	79	133.42 ± 12.57	45	125.71 ± 14.32	0.002	d = 0.583
Platelet (/mL)	79	229 (197.5–291)	45	243 (202–285)	0.682	d = 0.074
Neutrophil (%)	79	59.95 ± 11.51	45	65.46 ± 12.84	0.015	d = 0.459
Lymphocyte (%)	79	28.52 ± 10.35	45	23.47 ± 10.81	0.011	d = 0.48
Erythrocyte sedimentation rate (mm/h)	79	20 (14–38)	45	28 (12–52)	0.260	d = 0.203
IgA (g/L)	80	1.95 (1.5–2.7)	45	2.1 (1.6–2.8)	0.088	d = 0.31
IgG (g/L)	80	10.2 (7.98–11.6)	45	10.6 (8.5–16.5)	0.089	d = 0.309
IgM (g/L)	78	1.13 (0.66–1.84)	45	1.1 (0.71–1.65)	0.983	d = 0.004
c3 (g/L)	80	1.4 (1.25–1.53)	45	1.21 (1.07–1.36)	<0.001	d = 0.877
c4 (g/L)	80	0.27 (0.23–0.31)	45	0.22 (0.17–0.27)	0.003	d = 0.549
CH50 (CH50/mL)	79	73 (59.5–83)	44	76.5 (63.5–82)	0.969	d = 0.007
anti-Ro/SS-A+	80	40 (50)	45	25 (55.56)	0.551	w = 0.053
anti-La/SS-B+	80	26 (32.5)	45	16 (35.56)	0.728	w = 0.031
Rheumatoid factor +	80	38 (47.5)	45	30 (66.67)	0.039	w = 0.185

Abbreviations: GFR: glomerular filtration rate, AST: aspartate transaminase, AP: alkaline phosphatase, GGT: gamma glutamil transferase, ALT: alanine transaminase, CRP: C-reactive protein, LDH: lactate dehydrogenase, IgA: Immunoglobulin A, IgG: Immunoglobulin G, IgM: Immunoglobulin M, c3: complement 3, c4: complement 4, CH50: 50% hemolytic complement activity, SS-A: anti-Sjögren’s-syndrome-related A antibody, SS-B: anti-Sjögren’s-syndrome-related B antibody.

**Table 4:** Differences in pharmacological management between the two groups.

	Obese, *n* (%)	Non-Obese, (*n*%)	*p*-Value	Effect-Size (w)
DMARD	47 (58.75)	32 (71.11)	0.169	0.123
Steroid	31 (38.75)	26 (57.78)	0.040	0.183
Statin	35 (43.75)	10 (22.22)	0.016	0.215

Abbreviation: DMARD: disease-modifying antirheumatic drug.

**Table 5:** Relationship between obesity (predictor) and ESSDAI (dependent) according to multiple linear regression.

	Unstandardized		Standardized	t	*p*-Value	95% Confidence Interval for B	
	B	Standard Error	β			Lower Bound	Upper Bound
(Constant)	−1.388	2.587		−0.537	0.593	−6.527	3.751
Obesity	−1.618	0.971	−0.172	−1.666	0.099	−3.548	0.311
Steroid	3.513	0.979	0.376	3.588	0.001	1.568	5.457
Statin	−0.018	0.955	−0.002	−0.018	0.985	−1.915	1.880
anti-Ro/SS-A+	0.436	1.019	0.048	0.428	0.670	−1.588	2.460
anti-La/SS-B+	0.348	1.166	0.036	0.298	0.766	−1.969	2.665
Rheumatoid factor +	0.072	0.985	0.008	0.073	0.942	−1.885	2.029
CRP (mg/L)	−0.054	0.058	−0.086	−0.932	0.354	−0.168	0.061
LDH (U/L)	0.022	0.009	0.235	2.528	0.013	0.005	0.038
Triglyceride (mmol/L)	−0.177	0.774	−0.024	−0.229	0.819	−1.714	1.359
Cholesterol (mmol/L)	0.061	0.369	0.017	0.166	0.869	−0.672	0.795

Abbreviations: SS-A: anti-Sjögren’s-syndrome-related A antibody, SS-B: anti-Sjögren’s-syndrome-related B antibody, CRP: C-reactive protein, LDH: lactated dehydrogenase.

## Data Availability

Data available from the authors upon request.
